# Ethics in Veterinary Practice

**Published:** 1881-04

**Authors:** 


Ethics in Veterinary Practice.—It is the custom of some veterinarians to advertise themselves, with much exuberance of language, in various agricultural, sporting, or stock journals.

There is no code in veterinary medicine which directly forbids this, but it is looked down upon by our best practitioners, and justly, as being a thing unworthy of the profession.

We would remind persons who are inclined to do this sort of thing, that it is a practice which, in human medicine, by common consent, stamps the man as a charlatan. Veterinarians, therefore, who follow it lay themselves open to the same imputation.

Advertising one's self is, of course, not an actual crime; but there is a rational basis to the feeling of the better men against it. A man who advertises his groceries or dry goods, advertises things which are quite apart from his own individuality. But a person who announces that he is the most skilful of surgeons in Dakota, or the greatest curer of spavins in all New England, or a graduate of more foreign and domestic colleges than anyone else in the United States—such a person thrusts his personality upon the public in a most offensive way. He lauds himself, which is bad, at the expense of his brother, which is worse. There is also very likely to be something in such advertisements which is not strictly true. It is hard to draw the limits when announcing one's own praise. A question of morals, therefore, comes in here. Apart from this, it is perhaps only a question of good and bad taste, of decent modesty or vulgar pretension.

It is a credit to the veterinary profession, after all, that advertising exists as little as it does. We trust it will not increase. Its decline and absence will mark a growth in the scientific standing of veterinary medicine, and in the respect felt for it by its practitioners.

To those who contend for advertising, we would admit this. There may be occasions when the putting a plain card announcement in local journals is justifiable. Itinerancy among veterinary surgeons is at present, at times, almost unavoidable; and for these some card announcement is perhaps allowable.

